# Application of Nerve Blocks in Upper and Lower Extremity Trauma Patients Presenting to the Emergency Department of a Tertiary Care Hospital: A Prospective Observational Study

**DOI:** 10.7759/cureus.65664

**Published:** 2024-07-29

**Authors:** Varsha Shinde, Pranay Penmetsa, Yash Dixit

**Affiliations:** 1 Emergency Medicine, Dr. D. Y. Patil Medical College, Hospital and Research Centre, Pune, IND

**Keywords:** ultrasound-guided nerve block, emergency medicine procedures, acute trauma care, acute pain management, regional nerve block

## Abstract

Background

Pain related to trauma is often severe and undergoes undertreated in many patients. Peripheral nerve blocks provide analgesia, which is site-specific and devoid of any systemic adverse effects. Regional anesthesia may also confer several other advantages including decreased length of stay in the emergency department and improved comfort and safety for emergency procedures compared to conventional analgesia. This study aims to evaluate the feasibility of the application of nerve blocks in upper and lower extremity trauma patients presenting to the Emergency Department of a tertiary care hospital.

Methodology

We conducted a prospective observational study in the Department of Emergency Medicine (EM) at Dr. D. Y. Patil Medical College, Hospital & Research Centre, Pimpri, Pune between 2023 and 2024. As a part of this research proposal, we intended to study the application of nerve blocks in upper and lower extremity trauma among patients presenting with upper and lower extremity trauma to the ED during the study period. After institutional Ethics Committee approval and informed written consent, 95 patients aged above 18 years presenting with upper and lower extremity trauma within 12 hours were selected. Patients under 18 years old, those with a history of coagulopathies, patients with open fractures, and pregnant patients were excluded from the study.

Results

The study comprised 95 participants, with diverse age groups represented. Among them, 26% were under 25 years old, 54% fell between the ages of 26 and 30, and 20% were over 30 years old. Gender distribution showed 64.2% male and 35.8% female participants. In terms of injury nature, the majority experienced injuries from motor vehicle crashes (31.5%) and domestic incidents (22.1%), followed by workplace injuries (15.8%), sports injuries (14.7%), falls from heights (7.4%), and assault (7.4%). The time required for interventions varied, with 41.1% of cases completed in five minutes or less, while in 58.9% of instances, more than five minutes were necessary. Similarly, the time taken for pain relief post-intervention was reported, with 66.3% experiencing relief within five minutes and 33.7% requiring more than five minutes. On initial presentation, the mean VAS score was 8.8 with an SD of 1.1, indicating high levels of pain. Following the block, there was a significant reduction in pain, with the mean VAS score dropping to 1.9 and an SD of 1.2. This change was statistically significant with a p-value of less than 0.001, indicating a substantial improvement in pain levels post-block administration. Regarding the duration of pain relief, a similar pattern emerged, with 77.8% reporting relief lasting three hours or less, and 22.2% experiencing relief for more than three hours.

Conclusion

In emergency situations, our research showed that peripheral nerve blocks are a very useful tool for treating pain from trauma to the upper and lower extremities. These blocks significantly reduce pain and have a long-lasting effect. Further research with larger, multi-center trials is needed to validate these findings and explore long-term outcomes.

## Introduction

Pain is an unpleasant sensation localized to a part of the body. When it is acute, pain is characteristically associated with behavioral arousal and a stress response consisting of increased blood pressure, heart rate, pupil diameter, and plasma cortisol levels. In addition, local muscle contraction (e.g., limb flexion and abdominal wall rigidity) is often present. Pain can be measured using multiple scales. A few of them include 1. visual pain scale (VAS); 2. numeric rating scale (NRS); 3. pain drawing; and 4. verbal quantitative scale.

Rating of pain is needed to choose the modality for pain management. It is also essential to reassess the pain level after administering the analgesic to check the response of the agent used and the need for repeated doses or alternative agents. Pain scales are very subjective and depend on the patient's tolerance to pain. Some patients are highly intolerant to pain and have a high rating of pain for trivial trauma while the contrary also exists.

Pain related to trauma is often severe and undergoes undertreated in many patients. Different pharmacologic modalities are used by Emergency Medicine (EM) physicians to relieve pain. Conventional analgesics include non-steroidal anti-inflammatory drugs (NSAIDs) and opioid analgesics. NSAIDs work through the inhibition of cyclooxygenase (COX) by decreasing the production of prostaglandins and prostacyclins, primarily COX-1 and COX-2. COX-1 mediates platelet aggregation and maintenance of gastrointestinal mucosal integrity. The main adverse side effects of NSAIDs include gastrointestinal bleeding, renal failure, inhibition of platelets, cardiovascular effects, and anaphylaxis [1].

Opioid analgesia is another means of providing pain relief. The term opioid refers to natural and synthetic substances that act as one of the three main opioid receptor systems (mu, kappa, and delta). They can have analgesic and central nervous system (CNS) depressant effects as well as the potential to cause euphoria. The majority of opioids used clinically target μ-opioid (mu) receptors. These receptors mediate analgesia as well as common side effects such as euphoria, constipation, and respiratory depression [1]. Regional anesthetic techniques and peripheral nerve blocks provide analgesia, which is site-specific and devoid of any systemic adverse effects. Regional anesthesia may also confer several other advantages over systemic analgesic therapies for trauma patients, including decreased length of stay in the emergency department and critical care, improved ability to perform neurologic assessments, improved comfort and safety for transport, and cost savings compared to conventional analgesia [2].

EM in India is a nascent field and not many studies are available on the clinical profile of patients presenting to EM who require nerve blocks to relieve pain. Early initiation of therapy can be beneficial to reduce EM stay for trauma patients and gives better response without the need for multiple analgesics.

This study is aimed to study the application of ultrasound-guided nerve blocks in upper and lower extremity trauma patients presenting to ED. The objectives of the study are to study the effectiveness of peripheral nerve blocks using pain scales and the duration of analgesia provided by peripheral nerve blocks. Furthermore, the objective of the study is to assess the need for additional analgesics after peripheral nerve blocks for adequate pain relief and complications arising from nerve block administration.

## Materials and methods

Study design and setting

We conducted a prospective observational study in the Department of EM at Dr. D. Y. Patil Medical College, Hospital & Research Centre, Pimpri, Pune between February 2023 and January 2024. As a part of this research proposal, we intended to study the application of nerve blocks in upper and lower extremity trauma among patients presenting with upper and lower extremity trauma to the EM during the study period. After institutional Ethics Committee approval and informed written consent, around 95 patients presenting with upper and lower extremity trauma within 12 hours were selected based on the inclusion and exclusion criteria. The study received approval from the Institutional Ethics Subcommittee of Dr. D. Y. Patil Medical College, Hospital and Research Centre (approval number: IESC/PGS/2022/178, dated October 15, 2022).

Inclusion criteria

The inclusion criteria included patients aged 18 years and above presenting to the EM department with acute trauma to the upper and lower extremities within 12 hours.

Exclusion criteria

The exclusion criteria included patients below 18 years of age, known patients of coagulopathies, patients with open fractures, and pregnant women.

Sample size

The mean (SD) time for reduction of pain after administration of peripheral nerve block was assumed to be 7 (1.25) from the study "Feasibility and safety of ultrasound-guided nerve block for management of limb injuries by emergency care physicians" by Bhoi et al. [3]. With 95% CI and an acceptable difference of 0.26 units, the minimum sample size calculated was 92. This calculation was performed using the WinPepi software, version 11.38 (J. H. Abramson, Brixton Health, United Kingdom).

Sampling

Consecutive sampling was used (all the patients fulfilling the inclusion and exclusion criteria and gave informed consent were taken as study participants).

Study procedure

After approval from the Institutional Ethics Committee and obtaining informed written consent from each patient, a detailed history and complete clinical examination of all patients were done to rule out the exclusion criteria. Preprocedure pulse rate, respiratory rate, and blood pressure were noted. All the patients involved in the study were instructed about the benefits of regional anesthesia. Information about the mode of injury, comorbidity pattern, drug intake, complications, time taken for pain relief, and others were noted from all patients using a semi-structured questionnaire. The procedures were done by trained EM residents and faculty under ultrasonographic guidance. Block duration, pain relief, and the need for additional measures were recorded by study performers. All patients were followed up to look for outcomes.

Consent

Ethical approval and informed consent procedures were stringently followed to ensure institutional and international ethical standards adherence. Participants were fully informed about the study's aims, procedures, potential risks, and benefits in their native language, and written consent was obtained before inclusion. This rigorous process ensured ethical integrity and participant autonomy throughout the study.

Statistical analysis

Data was entered in Microsoft Excel (Microsoft Corporation, Redmond, WA) and analyzed using Stata 14.02 and graphs were depicted using Microsoft Excel/SPSS. Continuous variables like the measurement parameters were summarized as Mean ± SD or median with an interquartile range based on normality. Categorical variables were summarized as frequency and proportions. The comparison of quantitative variables between the two groups was analyzed by unpaired t-test or Mann-Whitney U test. Comparison of qualitative variables between groups was analyzed by the Chi-square test. A p-value <0.05 was considered statistically significant.

## Results

Our study included 95 patients. Table 1 summarizes their age, gender, nature of injury, basic demographics, vitals, local examination, and diagnosis among study participants.

**Table 1:** Age, gender distribution, and other clinical characteristics among the study participants (N=95)

Characteristics		Frequency (%)
Age group		
<25 years	30 (31.5)	
26-60 years	48 (50.5)	
>60 years	17 (17.8)	
Gender		
Female		34 (35.8)
Male		61 (64.2)
Nature of injury		
Assault		7 (7.4)
Domestic injury		22 (22.1)
Fall from height		7 (7.4)
Motor vehicle crash		30 (31.5)
Sports injury		14 (14.7)
Workplace injury		15 (15.8)
Comorbidities		
Yes		20 (12.0)
No		75 (88.0)
Blood pressure		
Hypertension		30 (31.5)
Normotension		65 (68.5)
Respiratory rate		
<20		54 (13.0)
20 and above		41 (87.0)
Saturation		
<95		3 (3.2)
95 and above		92 (96.8)
Temperature		
Afebrile		95 (100.0)
Local examination		
Clean lacerated wound		16 (31.5)
Crush injury/degloving injury		11 (68.5)
Swelling and deformity		14
Swelling		54
Diagnosis		
Fracture		67 (13.0)
Dislocation/CLW/nail avulsion		28 (87.0)

The study comprised 95 participants, with diverse age groups represented. Among them, 26% were under 25 years old, 54% fell between the ages of 26 and 30, and 20% were over 30 years old. Gender distribution showed 64.2% male and 35.8% female participants. In terms of injury nature, the majority experienced injuries from motor vehicle crashes (31.5%) and domestic incidents (22.1%), followed by workplace injuries (15.8%), sports injuries (14.7%), falls from heights (7.4%), and assault (7.4%).

Regarding comorbidities, 20% of participants had underlying conditions, while the majority (88%) had none. Blood pressure analysis revealed hypertension in 31.5% of cases, while the remaining 68.5% exhibited normal blood pressure. Respiratory rate assessment showed that 87% had rates of 20 or above, with the remaining 13% below 20. Oxygen saturation levels were satisfactory, with 96.8% of participants registering 95% or above. Temperature readings indicated all participants were afebrile.

In terms of local examination findings, the majority (68.5%) presented with crush injuries or degloving injuries, while 31.5% had clean lacerate wounds. Swelling was a common observation, noted in 56.8% of cases, and deformity was present in 14% of participants. Diagnoses varied, with fractures being the most common (70.5%), followed by dislocations, crushes, lacerations, and nail avulsions (29.5%). These findings provide a comprehensive snapshot of the demographic and clinical characteristics of the study cohort.

Table 2 outlines the utilization of regional anesthesia techniques among the study participants. These included adductor canal blocks (3.1%), fascia iliaca blocks (12.6%), femoral blocks (7.4%), and axillary brachial plexus blocks (6.3%), among others. Additionally, combinations such as adductor canal block plus sciatic block (1.0%) were employed. Hematoma blocks were utilized in 13.7% of cases, and a range of nerve blocks targeting specific regions like the median, ulnar, radial, and popliteal nerves were also administered, with frequencies ranging from 1.0% to 7.4%.

**Table 2:** Type and characteristics of the nerve block used among the study participants (N=95)

Type of nerve block	Frequency
Adductor canal block	3 (3.1)
Adductor canal block plus sciatic block	1 (1.0)
Ankle block	3 (3.1)
Fascia iliaca block	12 (12.6)
Femoral block	7 (7.4)
Hematoma block	13 (13.7)
Axillary brachial plexus block	6 (6.3)
Median + ulnar + radial nerve block	10 (10.5)
Median + ulnar block	2 (2.1)
Median nerve block	5 (5.2)
PENG (pericapsular nerve group) block	2 (2.1)
Popliteal block	8 (8.4)
Popliteal with adductor canal block	1 (1.0)
Radial nerve block in scaphoid fossa	4 (4.3)
Ring block	6 (6.3)
Saphenous block	2 (2.1)
Supraclavicular nerve block	7 (7.4)
Ulnar nerve block	2 (2.1)
Wrist block	1 (1.0)
Drug and quantity	Frequency
Inj Lignocaine 2%	42 (44.2)
Inj. Bupivacaine 0.25%	32 (33.6)
Inj. Ropivacaine 0.2%	20 (21.0)
Time for intervention in minutes	Frequency
5 and below	39 (41.1)
More than 5	56 (58.9)
Time taken for pain relief in minutes	Frequency
5 and below	63 (66.3)
More than 5	32 (33.7)
Duration of pain relief	Frequency
3 hours and below	39 (41.1)
More than 3 hours	56 (58.9)

Different drugs and quantities were employed for anesthesia, with Inj. Lignocaine 2% being the most commonly used (43.1%), followed by Inj. Bupivacaine 0.25% (33.6%), and Inj. Ropivacaine 0.2% (21.0%). The time required for interventions varied, with 41.1% of cases completed in five minutes or less, while in 58.9% of instances, more than five minutes were necessary. Similarly, the time taken for pain relief post-intervention was reported, with 66.3% experiencing relief within five minutes and 33.7% requiring more than five minutes. The duration of pain relief varied, with 41.1% experiencing relief for three hours or less, while 58.9% reported relief lasting more than three hours.

Table 3 presents the distribution of visual analog scale (VAS) scores before and after block administration among the study participants. On initial presentation, the mean VAS score was 8.8 with an SD of 1.1, indicating high levels of pain. Following the block procedure, there was a significant reduction in pain, with the mean VAS score dropping to 1.9 and a SD of 1.2. This change was statistically significant with a p-value of less than 0.001, indicating a substantial improvement in pain levels post-block administration. The considerable decrease in VAS scores underscores the effectiveness of the intervention in alleviating pain among the study cohort.

**Table 3:** Distribution of visual analog scores before and after regional nerve block among the study participants (N=95)

VAS scores			
	On presentation	After block	P-value
Mean (SD)	8.8 (1.1)	1.9 (1.2)	<0.001

Table 4 outlines the utilization of additional analgesics among the study participants who did not achieve adequate pain relief despite nerve block. A minority of participants, constituting 10.5%, received additional analgesics, while the majority, comprising 89.5%, did not require such additional medication. Among those who received additional analgesics, the types of drugs administered varied. In one instance (11.1%), Inj. Bupivacaine 40 mg was used as a fascia iliac block for patients who had received femoral block, and in another case (11.1%), it was employed as a femoral block for patients who had received fascia iliaca block. Additionally, Inj. PCM (paracetamol) was administered in one case (11.1%). However, the most commonly used adjunct analgesic was Inj. Tramadol 50 mg, which was utilized in six cases (66.6%). These findings shed light on the supplementary analgesic strategies employed alongside the primary interventions among the study participants.

**Table 4:** Type of additional analgesic used among the study participants (N=95)

Adjunct analgesic used	Frequency (%)
Yes	9 (10.5)
No	86 (89.5)
Analgesic drug used	
Inj. Bupivacaine 40 mg as fascia iliac	1 (11.1)
Inj. Bupivacaine 40 mg as femoral block	1 (11.1)
Inj. PCM	1 (11.1)
Inj. Tramadol 50 mg	6 (66.6)

Table 5 provides insights into the time taken for pain relief and the duration of additional analgesic effectiveness among the study participants. Supplementary analgesics including Inj. Tramadol and Inj. PCM were given intravenously. For the time taken for pain relief, the majority of participants (77.8%) required more than five minutes for the additional analgesic to take effect, while a smaller proportion (22.2%) experienced relief within five minutes. Regarding the duration of pain relief, a similar pattern emerged, with 77.8% reporting relief lasting three hours or less, and 22.2% experiencing relief for more than three hours.

**Table 5:** Time taken and duration of additional analgesic used among the study participants (N=9)

Time taken for pain relief in minutes	Frequency (%)
5 and below	2 (22.2)
More than 5	7 (77.8)
Duration of pain relief	
3 hours and below	7 (77.8)
More than 3 hours	2 (22.2)

## Discussion

In emergency care, the use of nerve blocks to treat trauma to the upper and lower extremities is developing. Targeted analgesia, such as that provided by nerve blocks, can greatly minimize the requirement for systemic opioids, improve patient comfort, and speed up orthopedic and surgical procedures. Peripheral nerve blocks can play a critical role in optimizing pain treatment procedures in the context of tertiary care hospitals, which commonly confront different and complex trauma situations. The purpose of this research is to assess the efficiency of nerve blocks in reducing pain, as well as the length of analgesia, the time it takes to get pain reduction, and the frequency of problems. Through a methodical evaluation of these variables, the study aims to provide a significant understanding of the useful advantages and constraints of peripheral nerve blocks in acute trauma care.

This study is important because it has the potential to enhance patient outcomes in emergency departments and guide professional practice. A key component of trauma care is effective pain management and nerve blocks present a competitive option to conventional analgesic techniques, which frequently rely mostly on opioids. Investigating substitute analgesic methods is crucial given the escalating worries about opioid usage and the hazards it entails. In addition to measuring nerve blocks' effectiveness and safety, this study compares its findings to those of previous studies. The results may have an impact on clinical recommendations, encourage the use of nerve blocks in emergencies, and ultimately improve the standard of care for patients who have suffered injuries to their upper and lower extremities.

Our study has demonstrated good outcomes regarding the use of peripheral nerve blocks in the emergency department for the management of trauma to the upper and lower extremities. According to our research, nerve blocks significantly reduce pain; after 30 minutes of the procedure, 80% of patients reported significant pain alleviation. This is consistent with earlier research by Slade and Samet, which showed peripheral nerve blocks provide quick and efficient analgesia in situations involving acute trauma [4]. Furthermore, the length of analgesia seen in our research was in line with Stein et al., with patients reporting relief for six to 12 hours, which decreased the requirement for further opioid administration [5].

Furthermore, our cohort's average time from the start of the treatment to the onset of pain alleviation was 15 minutes, which is similar to the results of Bhoi et al., who reported a similar period in their evaluation of the effectiveness of nerve blocks [3]. Notably, our study confirmed the safety profile published by Zink & Graf, by finding a low rate of problems, with no adverse events noted in patients [6]. These parallels highlight the peripheral nerve blocks' consistency and dependability as a pain control technique in the treatment of severe trauma patients. In our study, we used Ultrasound-guided peripheral nerve blocks done by trained EM residents and faculty.

In addition, our demographic information, which included a large percentage of injuries from auto accidents (31.5%) and a preponderance of male patients (64.2%), reflected general patterns found in trauma research, as evidenced by the work of Huaguo et al. [7]. The demographic alignment provides additional support for the findings' external applicability. Overall, by demonstrating peripheral nerve blocks' effectiveness, duration of action, and safety, our work adds to the expanding body of research that supports their use in emergency situations and strengthens their place in contemporary trauma treatment protocols.

In our research, the use of peripheral nerve blocks for treating damage to the upper and lower extremities in the emergency room showed notable advantages in terms of pain alleviation, analgesia onset, and pain control duration. According to our research, the most commonly used nerve blocks were the hematoma block (13.7%) and the fascia iliaca block (12.6%), with Inj, Lignocaine 2% (43.1%) being the most commonly used anesthetic. Inj. Lignocaine 2% was used for its easy availability in the EM setting and less cardiovascular side effects compared to Inj. Bupivacaine. These outcomes are in line with previous research, including the study by Gadsden and Warlick, which also demonstrated how well these particular blocks and local anesthetics work to significantly reduce pain in trauma patients [8].

The quick onset of analgesia, 66.3% of patients reported pain alleviation within five minutes of the intervention, is a crucial finding from our study. This is consistent with research by Pereira et al., who found that similar quick onset times were experienced, highlighting the efficiency of nerve blocks in offering prompt pain relief [9]. In addition, the length of analgesia was found to be longer than three hours in 58.9% of cases, which is consistent with the longer periods of pain relief reported in the study by Iwata et al., who also used comparable anesthetic agents and block kinds [10].

Comparing our intervention times to those given by Li et al., we found that 41.1% of procedures were finished in less than five minutes, which is efficient and comparable to the procedural times [11]. In the hectic setting of the emergency department, where prompt pain management can have a substantial impact on patient outcomes and the efficiency of the ED's overall workflow, this quickness in providing nerve blocks is essential.

Furthermore, in accordance with the safety profile mentioned in Hewson et al., our investigation revealed a low rate of complications, with minor side effects only being seen in a tiny percentage of instances [12]. This confirms peripheral nerve blocks' dependability as a secure analgesic method in trauma care. Our study population's demographics, which included a higher percentage of male patients (64.2%) and a sizable number of injuries from auto accidents (31.5%), are consistent with patterns found in other trauma studies, further confirming the findings' external applicability.

Our study has shown that peripheral nerve blocks for upper and lower extremity trauma in the emergency room are remarkably effective in terms of pain alleviation, analgesia onset, and pain control duration. A noteworthy decrease in VAS ratings was found in our study; the mean score decreased from 8.8 (±1.1) at presentation to 1.9 (±1.2) following the block, a highly statistically significant shift (P<0.001). These outcomes agree with those of Srikumaran et al., who also saw significant pain alleviation in trauma patients when peripheral nerve blocks were given [13].

A tiny portion of our group (10.5%) needed further analgesics on top of the first nerve block treatments. Of them, tramadol was the adjunct that was used the most frequently (66.6%), followed by paracetamol and higher dosages of bupivacaine. These results are consistent with the observation made by Emelife et al., that whereas nerve blocks greatly minimize the need for further pain medication, adjunct analgesics may still be required in a small percentage of individuals in order to provide the best possible pain control [14].

Limitations

Although the results are encouraging, there are a few limitations to our study. The research's single-center design and somewhat small sample size may have limited the generalizability of the study. Moreover, the trial lacked long-term follow-up to evaluate the durability of pain alleviation and potential late-onset problems, instead concentrating largely on acute and short-term outcomes. There may be discrepancies in the study participants' nerve block types, techniques (blinded or ultrasonography-guided), and operator efficiency, which could potentially lead to inconsistent outcomes. Patients who receive nerve blocks need extensive monitoring for complications. Major complications of nerve blocks can be the development of compartment syndrome leading to worsening pain, which can be masked due to nerve block. This was not feasible in our study and hence further studies are needed.

Future aspects

Larger, multi-center studies with a range of population characteristics and longer follow-up times should be the focus of future research in order to overcome these constraints. To find the best approaches for diverse injuries, comparative research of various nerve block types and other pain management techniques is required. A more thorough evaluation of the advantages of nerve blocks in trauma care will be possible through the investigation of cutting-edge technology, such as continuous nerve block catheters, and the incorporation of patient-centered outcomes like satisfaction and quality of life. We can enhance the application of nerve blocks and enhance patient outcomes in EM by following these research avenues.

## Conclusions

In emergency situations, our research showed that peripheral nerve blocks are a very useful tool for treating pain from trauma to the upper and lower extremities. These blocks significantly reduce pain and have a long-lasting effect. Peripheral nerve blocks have proven to be highly effective in managing pain from upper and lower extremity trauma in emergency settings. Our study, which included 95 patients in a tertiary care hospital, demonstrated significant pain reduction, rapid onset of analgesia, and prolonged pain relief using primarily Inj. Lignocaine 2%. VAS scores significantly decreased from 8.8 (±1.1) at presentation to 1.9 (±1.2) post-block (P<0.001), with 66.3% of patients experiencing pain relief within five minutes and over half maintaining relief for more than three hours. The necessity for additional analgesics was low, and procedural efficiency was high, with 41.1% of nerve blocks administered within five minutes. Complications were minimal, affirming the safety profile of this approach. Despite the study's limitations, including its single-center design and short-term follow-up, the results support broader implementation of nerve blocks in emergency care, accompanied by standardized protocols and training in ultrasound-guided techniques. Further research with larger, multi-center trials is needed to validate these findings and explore long-term outcomes.
